# Induction and requirement of gene expression in the anterior cingulate cortex and medial prefrontal cortex for the consolidation of inhibitory avoidance memory

**DOI:** 10.1186/1756-6606-4-4

**Published:** 2011-01-19

**Authors:** Yue Zhang, Hotaka Fukushima, Satoshi Kida

**Affiliations:** 1 Department of Bioscience, Faculty of Applied Bioscience, Tokyo University of Agriculture, Tokyo 156-8502, Japan; 2 Core Research for Evolutional Science and Technology, Japan Science and Technology Agency, Saitama 332-0012, Japan

## Abstract

**Background:**

Memory consolidation is a process to stabilize short-term memory, generating long-term memory. A critical biochemical feature of memory consolidation is a requirement for gene expression. Previous studies have shown that fear memories are consolidated through the activation of gene expression in the amygdala and hippocampus, indicating essential roles of these brain regions in memory formation. However, it is still poorly understood whether gene expression in brain regions other than the amygdala/hippocampus is required for the consolidation of fear memory; however, several brain regions are known to play modulatory roles in fear memory formation.

**Results:**

To further understand the mechanisms underlying the formation of fear memory, we first identified brain regions where gene expression is activated after learning inhibitory avoidance (IA) by analyzing the expression of the immediately early genes c-fos and Arc as markers. Similarly with previous findings, the induction of c-fos and Arc expression was observed in the amygdala and hippocampus. Interestingly, we also observed the induction of c-fos and Arc expression in the medial prefrontal cortex (mPFC: prelimbic (PL) and infralimbic (IL) regions) and Arc expression in the anterior cingulate cortex (ACC). We next examined the roles of these brain regions in the consolidation of IA memory. Consistent with previous findings, inhibiting protein synthesis in the hippocampus blocked the consolidation of IA memory. More importantly, inhibition in the mPFC or ACC also blocked the formation of IA memory.

**Conclusion:**

Our observations indicated that the formation of IA memory requires gene expression in the ACC and mPFC as well as in the amygdala and hippocampus, suggesting essential roles of the ACC and mPFC in IA memory formation.

## Background

To form long-term memory (LTM), short-term memory (STM) is stabilized through a process known as memory consolidation [1-3]. A critical biochemical feature of memory consolidation is a requirement for gene expression [3-8].

The expression of immediate-early genes (IEGs), such as c-fos, Arc, and Zif268, is regulated in a neural activity-dependent manner [9-16]. Therefore, the expression of IEGs has been used as a marker to identify brain regions that are activated in response to learning or memory retrieval [17-20]. Moreover, the activity-dependent expression of IEGs is thought to play a critical role in the formation of LTM [21-25]. Genetic inhibition of transcription factor cAMP-responsive element-binding protein (CREB)-mediated transcription, known as a master regulator of activity-dependent transcription, blocks the consolidation of LTM [26-30]. Consistently, recent genetic studies using mice have shown that the deletion of the Arc or c-fos gene, both of which are targets of CREB, led to the impairment of fear and spatial memories [31,32].

In fear conditioning tasks, a conditioned stimulus (CS; such as a context) is paired with an unconditioned stimulus (US; such as footshock). Animals learn this association of CS-US. Abundant studies have shown that the amygdala is a master brain area to learn fear and to form and express fear memories [33-36]. The hippocampus is also another critical area for the formation of several types of fear memory such as contextual fear and inhibitory avoidance (IA) memories [37-46]. Blocking protein synthesis in these brain areas around the time of training blocks the consolidation of fear memory [36,43,44,47-53].

In contrast, it remains poorly understood whether brain areas other than the hippocampus and amygdala play essential roles in the formation or consolidation of fear memory. However, other brain areas have been demonstrated to have critical roles in the regulation or modulation of fear memory. For example, the anterior cingulate cortex (ACC) plays a role in the early formation of contextual fear memory through the activation of the N-methyl-D-aspartate glutamate receptor (NMDAR) NR2B subunit [54,55] and the storage of long-lasting contextual fear memory [18]. On the other hand, the medial prefrontal cortex (mPFC including prelimbic (PL) and infralimbic (IL) regions) modulates the encoding of fear memory via dopamine transmission within the mPFC [56] and is required for the long-term extinction of fear memory in a gene expression-dependent manner [20,57,58]. Interestingly, previous studies have shown that the induction of IEGs or the activation of CREB were observed in the ACC, mPFC (PL and IL regions) [59,60], respectively, after fear conditioning, raising the possibility that these brain areas play essential roles in the formation phases of fear memory in a gene expression-dependent manner.

In this study, to understand the underlying mechanisms for the formation of fear memory, we first investigated brain regions where gene expression was activated after IA learning by analyzing the expression of IEGs. We then examined the roles of gene expression in the identified brain regions in the formation of IA memory.

## Results

### Requirement of protein synthesis for the formation of IA memory

**Figure 1:**
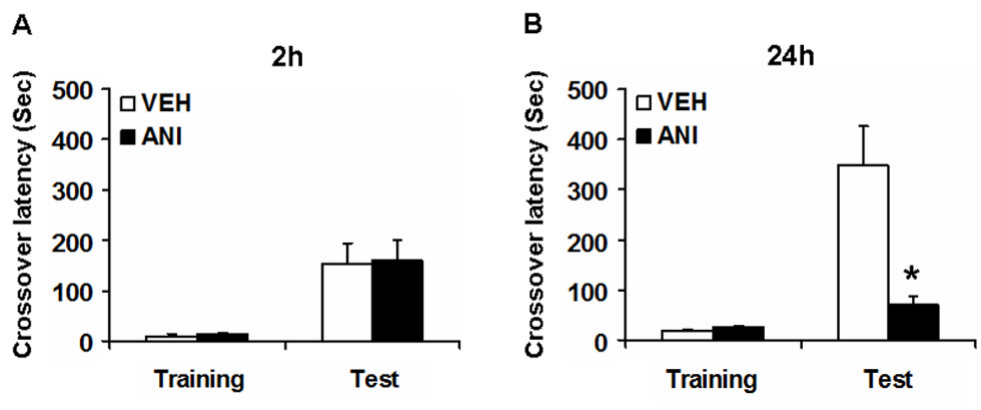
**Roles of protein synthesis in ST- and LT-IA memory**. (A) Effects of the injection of ANI on STM (VEH group, n = 8; ANI group, n = 7). (B) Effects of the injection of ANI on LTM (VEH group, n = 8; ANI group, n = 7). Error bars are SEM. *P < 0.05 compared with the VEH group.

We first tried to confirm the requirement of new gene expression for the formation of IA memory in our experimental conditions (Figure 1). Mice were first placed in the light compartment. Five seconds after mice entered into the dark compartment, a brief electrical footshock was delivered (training). Immediately after training, mice received a systemic injection of anisomycin (ANI; 150 mg/kg) or vehicle (VEH). Two or twenty-four hours later, mice were placed back in the light compartment and we assessed the crossover latency to enter the dark compartment (test). Two-way ANOVA followed by a *post hoc *Bonferroni's test revealed a significant effect of time (training vs. test), but not drug (VEH vs. ANI), and no significant time vs. drug interaction when 2 h-STM was assessed (time: F_1,26 _= 25.63, P < 0.05; drug: F_1,26 _= 0.029, P > 0.05; time vs. drug: F_1,26 _= 0.001, P > 0.05). Consistently, the *post hoc *Bonferroni's test revealed that the VEH and ANI groups showed comparable crossover latencies for the 2 h-STM (P > 0.05). In contrast, two-way ANOVA identified significant effects of time, drug, and a time vs. drug interaction when 24 h-LTM was assessed (time: F_1,24 _= 21.780, P < 0.05; drug: F_1,24 _= 11.140, P < 0.05; time vs. drug: F_1,24 _= 12.382, P < 0.05). Consistently, the *post hoc *Bonferroni's test revealed that the ANI group displayed a significantly shorter crossover latency tested at 24 h after the training than the VEH group (P < 0.05). These results indicated that the inhibition of protein synthesis blocked the formation of LTM without affecting STM, and confirmed previous observation that the systemic inhibition of protein synthesis impaired the retention of IA memory [61].

### Up-regulation of c-fos expression in distinct brain regions after IA training

To identify which brain regions were activated when IA memory is generated, we performed immunohistochemistry to measure the expression of the IEG c-fos whose expression is regulated by neuronal activity [9,15]. A conditioned group of mice received a footshock after they entered into the dark compartment in the training session (CS-US group). Unconditioned groups were treated similarly, except that they either did not receive a footshock in the dark compartment (CS group), or they directly received a footshock in the dark compartment in the training session without habituating to the light compartment (US group), or they were left undisturbed in their home cages throughout the experiments (home cage group). The unconditioned groups (CS and US) showed significantly shorter crossover latencies than the conditioned (CS-US) group during the test at 24 h after training (Additional file 1). The mice of CS-US, CS, and US groups were assessed c-fos expression at 90 min after training. The expression levels of c-fos in each brain region for each group were expressed as the ratio of averaged values in the home cage group. We analyzed the expression levels of c-fos in the amygdala [lateral (LA), basolateral (BLA), and central (CeA) regions], hippocampus [CA1, CA3, and dentate gyrus (DG) regions], mPFC [PL and IL regions], ACC, and the other cortical regions [visual cortex (VC), temporal cortex (TC), perirhinal cortex (PRh), and entorhinal cortex (EC) regions].

**Figure 2:**
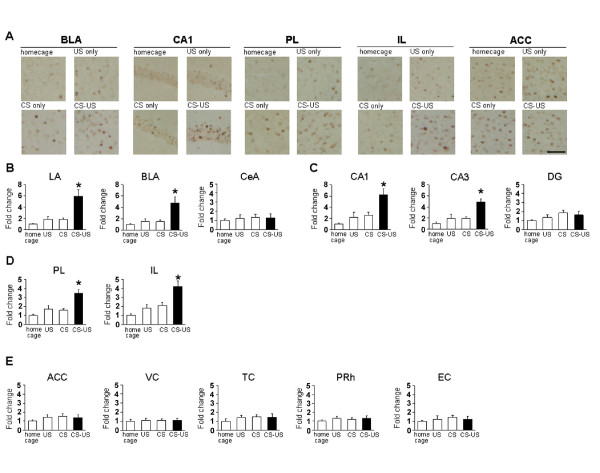
**c-fos expression in distinct brain regions after IA training**. (A) Representative immunohistochemical staining of PL, IL, BLA, CA1, and ACC c-fos-positive cells from the indicated mice. Scale bar, 50 μ m. (B) c-fos expression in the LA, BLA, and CeA regions of the amygdala (home cage, n = 8; US only, n = 6; CS only, n = 10; CS-US, n = 7). (C) c-fos expression in the CA1, CA3, and DG regions of the hippocampus (home cage, n = 7; US only, n = 6; CS only, n = 9; CS-US, n = 6). (D) c-fos expression in the prelimbic and infralimbic regions of the mPFC (home cage, n = 8; US only, n = 6; CS only, n = 10; CS-US, n = 7). (E) c-fos expression in the cortex regions (ACC: home cage, n = 8; US only, n = 6; CS only, n = 10; CS-US, n = 7; VC, TC, PRh, and EC: home cage, n = 6; US only, n = 5; CS only, n = 7; CS-US, n = 5). Error bars are SEM. *P < 0.05, compared with the other groups. c-fos expression for each group is expressed as the ratio of the home cage group to the other groups.

We first analyzed the expression levels of c-fos in the amygdala. Similarly with a previous finding that c-fos expression in the amygdala was induced after IA training [52], we observed the induction of c-fos expression in the LA and BLA regions, but not in the CeA region of the amygdala in the CS-US group after training in the IA task (Figure 2A, B). One-way ANOVA across the 4 groups (CS-US, CS, US, and home cage) revealed significant effects of group in the LA and BLA regions, but not in the CeA region (Figure 2B; LA: F_3,27 _= 13.762, P < 0.05; BLA: F_3,27 _= 9.168, P < 0.05; CeA: F_3,27 _= 0.261, P > 0.05). The *post hoc *Newman-Keuls test showed significantly higher levels of c-fos expression in the LA and BLA regions in the CS-US group than in the other groups (Figure 2B; P < 0.05).

We next analyzed the expression levels of c-fos in the hippocampus. Similarly with previous findings that c-fos expression was induced in the hippocampus after IA training [52,62,63], we observed the induction of c-fos expression in the CA1 and CA3 regions, but not in the DG region after training (Figure 2A, C). One-way ANOVA revealed significant effects of group in the CA1 and CA3 regions, but not in the DG region (Figure 2C; CA1: F_3,24 _= 8.296, P < 0.05; CA3: F_3,24 _= 11.203, P < 0.05; DG: F_3,24 _= 1.547, P > 0.05). The *post hoc *Newman-Keuls test showed significantly higher levels of c-fos in the CA1 and CA3 regions in the CS-US group than in the other control groups (Figure 2C; P < 0.05)

We next analyzed the expression levels of c-fos in the mPFC. Interestingly, the induction of c-fos expression was observed in the PL and IL regions after training (Figure 2A, D). One-way ANOVA revealed significant effects of group in the PL and IL regions (Figure 2D; PL: F_3,27 _= 13.417, P < 0.05; IL: F_3,27 _= 11.275, P < 0.05). The *post hoc *Newman-Keuls test showed significantly higher levels of c-fos in the PL and IL regions in the CS-US group than in the other control groups (Figure 2D; P < 0.05). These observations represent the first evidence showing the increased expression of c-fos in the mPFC after training for the IA task.

We finally analyzed the levels of c-fos expression in cortical regions. In contrast to the results described above, there was no increase in the expression of c-fos in the cortical regions analyzed in this study (Figure 2A, E). One-way ANOVA revealed no significant effect of group (Figure 2E; ACC: F_3,27 _= 0.907, P > 0.05; VC: F_3,19 _= 0.065, P > 0.05; TC: F_3,19 _= 0.524, P > 0.05; PRh: F_3,19 _= 0.433, P > 0.05; EC: F_3,19 _= 0.375, P > 0.05).

In summary, our anatomical analyses indicated the increased expression of c-fos in the amygdala, hippocampus, and mPFC after learning the IA, raising the possibility that gene expression in these brain regions contributes to the formation of IA memory.

### Up-regulation of Arc expression in distinct brain regions after IA training

To further characterize the brain regions where gene expression was activated following training in the IA task, we next measured the expression of another IEG, Arc, whose expression is regulated by neuronal activity [12-16]. Similarly with the analyses of c-fos expression, 4 groups of mice were analyzed in this experiment. The mice of CS-US, CS, and US groups were assessed Arc expression at 90 min after training.

**Figure 3:**
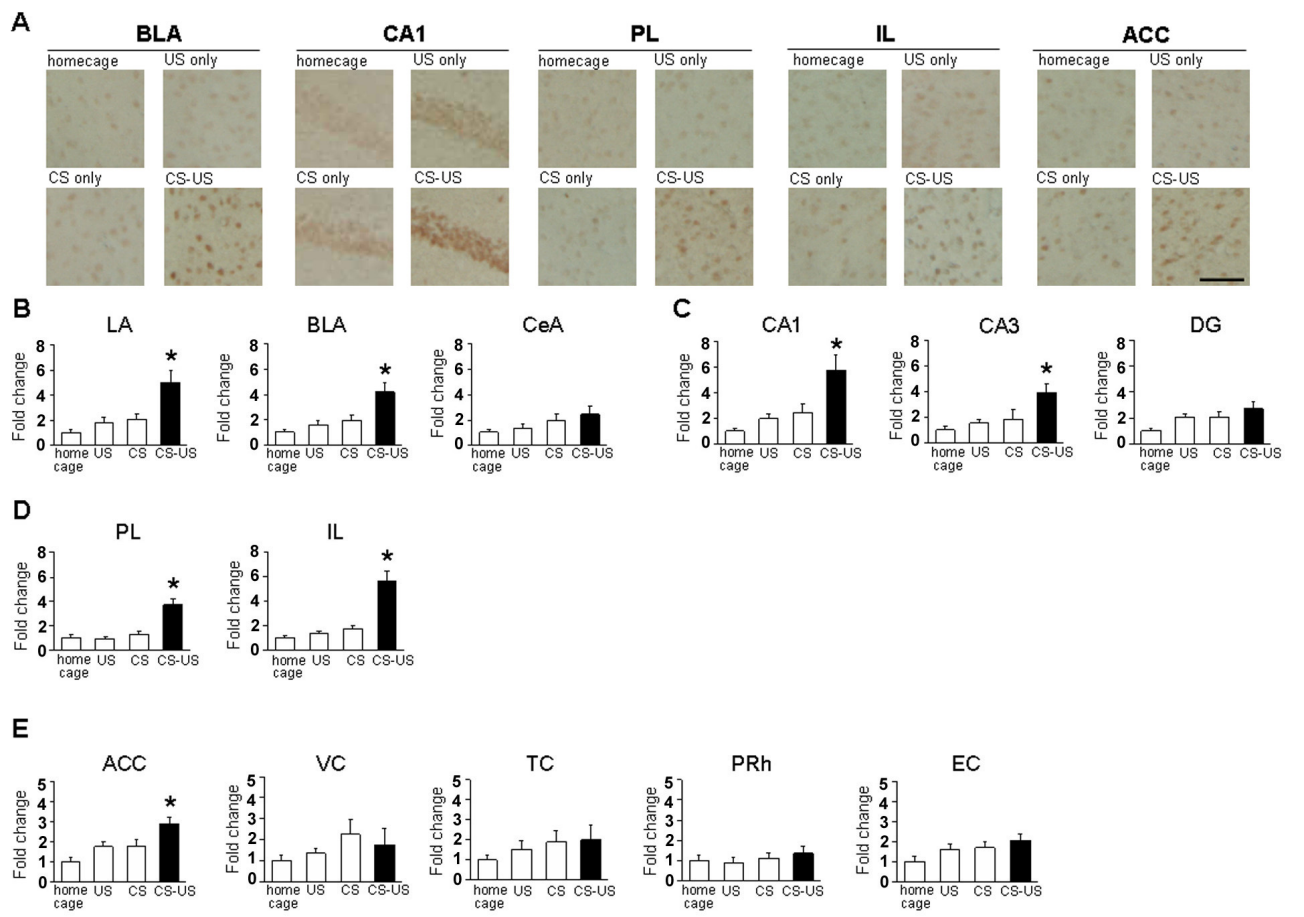
**Arc expression in distinct brain regions after IA training**. (A) Representative immunohistochemical staining of PL, IL, BLA, CA1, and ACC Arc-positive cells from the indicated mice. Scale bar, 50 μ m. (B) Arc expression in the LA, BLA, and CeA regions of the amygdala (home cage, n = 11; US only, n = 10; CS only, n = 12; CS-US, n = 10). (C) Arc expression in the CA1, CA3, and DG regions of the hippocampus (home cage, n = 11; US only, n = 11; CS only, n = 13; CS-US, n = 11). (D) Arc expression in the prelimbic and infralimbic regions of the mPFC (home cage, n = 13; US only, n = 12; CS only, n = 14; CS-US, n = 12). (E) Arc expression in the cortex regions (ACC: home cage, n = 13; US only, n = 12; CS only, n = 14; CS-US, n = 12; VC, TC, PRh, and EC: home cage, n = 10; US only, n = 9; CS only, n = 12; CS-US, n = 11). Error bars are SEM. *P < 0.05, compared with the other groups. Arc expression for each group is expressed as the ratio of the home cage group to the other groups.

Similarly with the results of c-fos expression (Figure 2B-D), IA training induced the expression of Arc in the LA and BLA regions of the amygdala, CA1 and CA3 regions of the hippocampus, and PL and IL regions of the mPFC, but not in the CeA region of the amygdala or the DG region of the hippocampus in the CS-US group (Figure 3A-D). One-way ANOVA revealed significant effects of group in the LA, BLA, CA1, CA3, PL, and IL regions, but not in the CeA or DG regions (Figure 3B-D; LA: F_3,39 _= 9.838, P < 0.05; BLA: F_3,39 _= 8.659, P < 0.05; CA1: F_3,42 _= 8.224, P < 0.05; CA3: F_3,42 _= 5.184, P < 0.05; PL: F_3,47 _= 16.799, P < 0.05; IL: F_3,47 _= 22.213, P < 0.05; CeA: F_3,39 _= 1.722, P > 0.05; DG: F_3,42 _= 0.017, P > 0.05). The *post hoc *Newman-Keuls test showed significantly higher levels of Arc expression in the LA, BLA, CA1, CA3, PL, and IL regions of the CS-US group than in the other groups (Figure 3B-D; P < 0.05).

More interestingly, in contrast to the results of c-fos expression (Figure 2E), the induction of Arc expression was observed in the ACC region of the CS-US group after training (Figure 3A, E). One-way ANOVA revealed a significant effect of group in the ACC region (F_3,47 _= 7.035, P < 0.05). The *post hoc *Newman-Keuls test showed a significantly higher level of Arc expression in the ACC of the CS-US group than in the control groups (Figure 3E; P < 0.05). These observations represent the first evidence showing the increased expression of Arc in the ACC after training in the IA task.

Consistent with the results of c-fos expression, there was no significant induction of Arc expression in the other cortical regions (Figure 3E). One-way ANOVA revealed no significant effect of group in the cortical regions (Figure. 3E; VC: F_3,38 _= 0.927, P > 0.05; TC: F_3,38 _= 0.736, P > 0.05; PRh: F_3,38 _= 0.498, P > 0.05; EC: F_3,38 _= 2.500, P > 0.05).

Taken together with the results of c-fos expression, these results indicated that the expression of IEGs (Arc and c-fos) were induced in the hippocampus, amygdala, and mPFC after learning the IA, suggesting critical roles for new gene expression in these brain regions in the formation of IA memory. Interestingly, we also observed that IA learning induced the expression of Arc in the ACC, suggesting a potential role of the ACC in the formation of IA memory.

### Requirement of protein syntheses in the mPFC and ACC for IA memory formation

Previous studies have shown that protein synthesis in the hippocampus and amygdala are required for the consolidation of IA memory [44,47,49,51-53,64]. Our analyses of gene expression after IA learning raised the possibility that new gene expression in brain regions (mPFC and ACC) other than the hippocampus and amygdala also contributes to the formation of IA memory. To examine this possibility, we tested the effects of inhibiting protein synthesis in the mPFC or ACC on the formation of IA memory. Mice were trained as described above (Figure 1). Immediately after training, mice received a micro-infusion of ANI (62.5 μg) or VEH into the hippocampus, mPFC, or ACC. Twenty-four hours later, crossover latency (test) was assessed in the mice. Cannula tip placements are shown in the supplementary data (Additional file 2). Only mice with cannula tips within the boundaries of the hippocampus, mPFC, or ACC were included in the data analysis.

**Figure 4:**
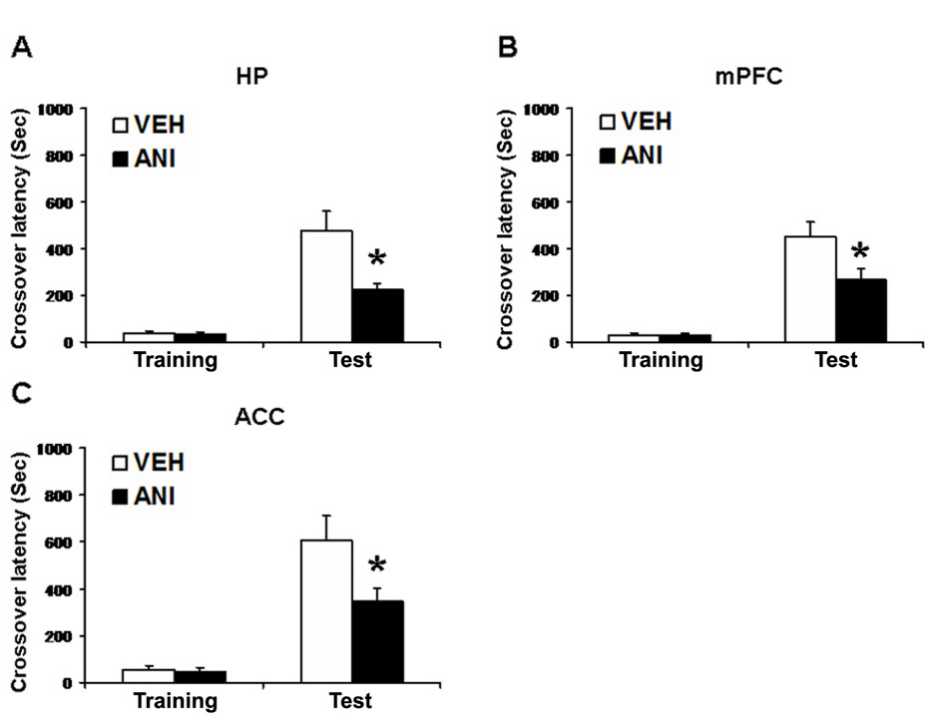
**Effects of protein synthesis inhibition in the hippocampus, mPFC, and ACC on the formation of IA memory**. (A) Effect of protein synthesis inhibition in the hippocampus (n = 9 per group). (B) Effect of protein synthesis inhibition in the mPFC (n = 8 per group). (C) Effect of protein synthesis inhibition in the ACC (n = 11 per group). Error bars are SEM. *P < 0.05 compared with the VEH group.

We first tried to confirm previous findings showing the requirement of new protein synthesis in the hippocampus for the formation of IA memory (Figure 4A). Two-way ANOVA identified significant effects of time, drug, and a time vs. drug interaction (time: F_1,32 _= 46.139, P < 0.05; drug: F_1,32 _= 7.735, P < 0.05; time vs. drug: F_1,32 _= 7.245, P < 0.05). The *post hoc *Bonferroni's test revealed that the ANI group displayed significantly shorter crossover latency at test than the VEH group (P < 0.05). These results indicated that the infusion of ANI into the hippocampus impaired the long-term IA memory and was consistent with previous findings [44,49,51,64], demonstrating that hippocampal protein synthesis is required for the formation of IA memory.

We next examined the effects of inhibiting protein synthesis in the mPFC or ACC (Figure 4B, C). Interestingly, similar to the results observed in the hippocampus, two-way ANOVA identified significant effects of time, drug, and a time vs. drug interaction (Figure 4B, C; mPFC, time: F_1,28_= 66.458, P < 0.05; drug: F_1,28_= 5.130, P < 0.05; time vs. drug: F_1,28 _= 5.215, P < 0.05; ACC, time: F_1,40 _= 47.517, P < 0.05; drug: F_1,40 _= 4.761, P < 0.05; time vs. drug: F_1,40 _= 4.222, P < 0.05). Consistently, the *post hoc *Bonferroni's test revealed that the ANI groups displayed significantly shorter crossover latencies at test than the VEH groups (P < 0.05). It is important to note that the infusion of ANI into the mPFC or ACC did not affect 2 h-ST-IA memory (Additional file 3) and that the inhibition of protein synthesis in the brain regions close to the mPFC or ACC (cannula tips failed to target on boundaries of the mPFC and ACC) did not affect LT-IA memory (Additional file 4). These results indicated that the infusion of ANI into the mPFC or ACC impaired the LT-IA memory without affecting ST-IA memory and that protein synthesis in the mPFC and ACC is required for the consolidation of IA memory, suggesting essential roles of the mPFC and ACC in IA memory.

## Discussion

A critical biochemical feature of memory formation is a requirement for gene expression [3-8]. In this study, we investigated brain regions where gene expression is activated after IA learning and the roles of gene expression in these brain regions in the formation of IA memory. To do this, we first showed that IA memory formation depends on new gene expression in our experimental conditions, confirming previous findings [61]. We next analyzed those brain regions where the expression of c-fos and Arc genes was induced because these 2 IEGs are regulated by neuronal activity [9-16] and are targets of CREB, which is required for the consolidation of LTM [26-30]. Importantly, genetic studies have shown that both IEGs are required for the formation of fear memories [31,32].

We observed that the expression of c-fos and Arc was induced in the hippocampus (CA1 and CA3 regions) and amygdala (LA and BLA regions) (Figure 2B, C). These observations were consistent with previous findings showing that CREB phosphorylation or c-fos expression in the hippocampus and amygdala are induced after IA training [52,62,63]. Furthermore, we observed that inhibiting protein synthesis in the hippocampus blocked the formation of LT-IA memory (Figure 4A), confirming previous findings that new gene expression in the hippocampus is required for the formation of LT-IA memory [44,49,51,64]. We did not observe the induction of c-fos and Arc expression in the DG region of the hippocampus and the entorhinal cortex following the IA learning, both of which are thought to play a role in encoding and/or storing context component of fear memory [35,65-67]. However, our results did not exclude that possibility that these brain regions play important roles in the formation of IA memory. It is important to examine the expression of other IEGs such as zif268 and the activation of signal transduction pathways including extracellular signal-regulated kinase (ERK) and CaMKII.

More importantly, we observed the induction of c-fos and Arc expression in the PL and IL regions of the mPFC (Figure 2D, 3D) and Arc expression in the ACC after IA training (Figure 3E). Similarly with the observations in the hippocampus, the blockade of protein synthesis in the mPFC or ACC inhibited the formation of LT-IA memory. Taken together with previous findings that inhibition of amygdaloid protein synthesis blocked the formation of LT-IA memory [47,52,53], our observations indicated that the formation of IA memory depends on new gene expression in the mPFC and ACC as well as in the hippocampus and amygdala.

The mPFC has been suggested to play modulatory roles in fear memory. For example, the mPFC modulates the encoding of fear memory via dopamine transmission within the mPFC [56] and the mPFC modulates the consolidation of IA memory by interacting with the basolateral amygdala [68]. In contrast, we showed that gene expression in the mPFC (PL and IL) is required for the consolidation of IA memory. The previous finding that c-fos expression is induced in the PL and IL regions of mPFC after contextual fear conditioning [59] supports our finding. Furthermore, other studies have shown that spatial, spatial working, object recognition, and long-term extinction memories are consolidated through the activation of gene expression or ERK in the mPFC [69-71]. Therefore, these observations, including ours, suggest that the mPFC plays essential roles in the consolidation of several types of memory.

Previous studies have shown that the ACC is involved in the regulation of fear memory [72-78]. The ACC modulates the consolidation of IA memory by interacting with the basolateral amygdala [79]. More importantly, blocking the activation of the NMDAR NR2B subunit in the ACC impaired contextual fear memory [54,55], indicating direct evidence for an essential role of the ACC in fear memory. Furthermore, the ACC plays a critical role in the storage and/or retrieval of remote contextual fear memory [18]. Even though these previous studies did not examine the effects of inhibiting protein synthesis, we showed that protein synthesis inhibition in the ACC blocked the consolidation of IA memory. Our study provides direct evidence indicating that the ACC plays an essential role in the consolidation of fear memory, suggesting that gene expression-dependent long-term neuroplastic changes in the ACC may be required for the formation of fear memory.

In this study, the induction of c-fos expression was observed in the mPFC, but not in the ACC, while the induction of Arc expression was observed in both regions. Nevertheless, protein synthesis inhibition in the mPFC or ACC blocked the formation of IA memory. These observations indicated that the mPFC and ACC display a comparable role in the formation of IA memory, although there are distinct mechanisms by which the expression of Arc and c-fos is induced. Similarly with our findings, previous studies also suggested the distinct regulation of Arc and c-fos expression. For example, experience of a novel taste led to an increase of c-fos mRNA expression in the amygdala and ACC and an increase of Arc mRNA expression in the hippocampus and ACC in mice [13]. Numerous studies have investigated the molecular mechanisms for the transcriptional regulation of the c-fos and Arc genes at the promoter level, and demonstrated the distinct structures of their promoters, regulatory elements, and transcription factors binding to these elements [16,80,81]. These different transcriptional regulatory mechanisms may be reflected by the distinct expression of c-fos and Arc genes after IA learning.

Our results suggest that IA memory is encoded in a broad network of cortical/subcortical regions including the mPFC, ACC, hippocampus and amygdala. Previous studies have shown direct connections between the amygdala and mPFC [82-88], amygdala and ACC [89-91], hippocampus and mPFC [92,93] and amygdala and hippocampus [88,94]. Especially, the amygdala interacts with many areas of the brain [34,95-97]. Therefore, the interactions among these brain regions may be required for the formation of IA memory. Indeed, previous studies have shown that the hippocampus, ACC and amygdala play critical roles in the formation of fear memory, respectively [75]. In addition, mPFC (PL and IL regions) may be required for some aspect of a decision making by which mice enter into the dark compartment associated with an aversive stimulus from the light compartment. Further studies are required to investigate the roles of these interactions among the hippocampus, amygdala, mPFC, and ACC in the formation of IA memory and to further understand the roles, functions, and molecular signatures of these brain areas in the formation of IA memory.

## Methods

### Animals

All experiments were conducted according to the *Guide for the Care and Use of Laboratory Animals, Japan Neuroscience Society *and the *Guide for the Tokyo University of Agriculture*. Male C57BL/6 mice were obtained from Charles River (Yokohama, Japan). Five or 6 mice were housed in cages, maintained on a 12 h light/dark cycle, and allowed *ad libitum *access to food and water. Mice were at least 8 weeks of age when tested. Testing was performed during the light phase of the cycle. Individual mice were used for all experiments. All experiments were conducted blind to the treatment conditions.

### Systemic injections

ANI (150 mg/kg, i.p.; Wako, Japan) was dissolved in saline (pH adjusted to 7.0-7.4 with NaOH) and administered to mice immediately after the behavioral manipulation. At this dose, ANI inhibits > 90% of protein synthesis in the brain during the first 2 h [98].

### Inhibitory avoidance test

The inhibitory avoidance test was performed as previously described [99]. Step-though inhibitory avoidance apparatus (OHARA Pharmaceutical, Tokyo, Japan) consisted of a box with separate light (15.5 × 12.5 × 11.5 cm) and dark (15.5 × 12.5 × 11.5 cm) compartments. The light compartment was illuminated by a fluorescent light (2500 lux). During the training session, each mouse was allowed to habituate to the light compartment for 30 s, and the guillotine door was raised to allow access to the dark compartment. Latency to enter into the dark compartment was considered as a measure of acquisition. Immediately after the mice entered the dark compartment, the guillotine door was closed. After 5 s, a footshock (0.2 mA) was delivered for a total period of 2 s. Memory was assessed at 2 or 24 h later as the crossover latency for the mice to enter into the dark compartment when replaced in the light compartment, as in training.

For the first experiment, we examined the effect of protein synthesis inhibition on STM and LTM. Mice received a systemic injection of saline or ANI (150 mg/kg) immediately after training, and memory was assessed at 2 (STM) or 24 (LTM) h later.

For the second experiment (immunohistochemistry), we examined the brain regions where gene expression was activated after training. Mice were divided into 4 groups: 1) CS-US group, mice were trained as described above; 2) CS group, mice received a training session in the absence of a footshock; 3) US group, mice were not allowed to habituate to the light compartment, but directly received a footshock in the dark compartment in the training session. The CS-US, CS, and US groups were anesthetized with Nembutal (750 mg/kg, i.p.) and perfused at 90 min after training. 4) Home cage group; mice were left undisturbed in their home cages throughout the experiment and anesthetized, as above, after they were taken from their home cages.

For the third experiment, we examined the effects of inhibiting protein synthesis in the hippocampus, mPFC, and ACC on IA memory. ANI was dissolved in vehicle solution (artificial cerebrospinal fluid (ACSF)), and adjusted to pH 7.0-7.4 with NaOH. Mice were trained as described above and received a microinfusion of ANI (62.5 μg) or ACSF immediately after training. Two or twenty-four hours after the training session, mice were once again placed in the light box, and crossover latency was assessed.

### Immunohistochemistry

After anesthetization, all mice were perfused with 4% paraformaldehyde. Brains were then removed, fixed overnight, transferred to 30% sucrose, and stored at 4°C. Coronal sections (30 μm) were cut in a cryostat. Sections were pretreated with 4% paraformaldehyde for 20 min and 3% H_2_O_2 _in methanol for 1 h, followed by incubation in blocking solution (PBS plus 1% goat serum albumin, 1 mg/mL BSA, and 0.05% Triton X-100) for 3 h. Consecutive sections were incubated with a polyclonal rabbit primary antibody for anti-c-fos (1:5000; Calbiochem) or anti-Arc (1:1000; Santa Cruz Biotechnology) in the blocking solution overnight. Subsequently, sections were washed with PBS and incubated for 3 h at room temperature with biotinylated goat anti-rabbit IgG (SAB-PO kit; Nichirei Biosciences), followed by 1 h at room temperature in the streptavidin-biotin-peroxidase complex (SAB-PO kit). Structures were anatomically defined according to the atlas of Franklin and Paxinos (1997) [100]. Quantification of c-fos- or Arc-positive cells in sections (100 × 100 μm) of the mPFC (bregma between +2.10 and +1.98 mm), ACC (bregma between +0.8 and +1.0), amygdala (bregma between -1.22 and -1.34 mm), dorsal hippocampus (bregma between -1.46 and -1.82 mm), VC (bregma between -3.88 and -4.00), TC (bregma between -3.88 and -4.00), PRh (bregma between -3.88 and -4.00), and EC (bregma between -3.88 and -4.00) were analyzed with a computerized image analysis system, as described previously (Winroof version 5.6 software; Mitani Corporation) [20]. Immunoreactive neurons were counted bilaterally with a fixed sample window across at least 3 sections by an experimenter blind to the treatment condition. The expression levels of c-fos and Arc for each group were expressed as the ratio of the averaged values in the home cage control group.

### Surgery and microinfusion of drug

Surgery was performed as described previously [19,20,101]. Under Nembutal anesthesia and using standard stereotaxic procedures, stainless steel guide cannulae (22 gauge) were implanted into the mPFC (2.7 mm, ± 0 mm, -1.6 mm), dorsal hippocampus (-1.8 mm, ± 1.8 mm, -1.9 mm), or ACC (1.0 mm, ± 0 mm, -1.6 mm). Mice were allowed to recover for at least 1 week after surgery. After this, they were handled for 1 week before the commencement of the IA test. Infusions into the hippocampus, mPFC, or ACC (0.5 μL) were made at a rate of 0.25 μL/min. This dose of locally infused ANI inhibits > 90% of protein synthesis for at least 4 h [102].

### Statistics

Data were analyzed with ANOVA. One-way and *post hoc *Newman-Keuls comparisons were used to analyze the expression of c-fos and Arc. Two-way ANOVA and *post hoc *Bonferroni's comparison were used to analyze the effects of time point, drug, and time point vs. drugs interaction. All values in the text and figure legends are means ± SEM.

## Competing interests

The authors declare that they have no competing interests.

## Authors' contributions

SK is responsible for the hypothesis development and overall design of the research and experiment, and supervised the experimental analyses. SK and YZhang co-wrote the manuscript. YZhang performed biochemical analyses. HF performed systemic injection analyses. YZhang and HF performed behavioral and micro-infusion analyses. All authors read and approved this manuscript.

## Supplementary Material

Additional file 1 **Crossover latencies of CS-US, CS and US groups**. CS and US groups showed significantly shorter crossover latencies than CS-US group during the test at 24 h after training **(**F_3,27 _= 22.390, P < 0.05**)**. CS-US, n = 11; CS only, n = 6; US only, n = 6. Error bars are SEM. *P < 0.05 compared with the unconditioned groups. Click here for file

Additional file 2 **Illustrating cannula tip placements in the hippocampus, mPFC, and ACC**. (A-C) Coronal drawing showing the placement of the cannula tip in the hippocampus (A), mPFC (B) and ACC (C). Only mice with needle tips within the boundaries of the hippocampus, mPFC, or ACC were included in the data analysis. VEH, vehicle; ANI, anisomycin. Click here for file

Additional file 3 **Effects of inhibiting protein synthesis in the mPFC and ACC on 2 h-STM**. (A) Effects of protein synthesis inhibition in the mPFC (VEH group, n = 11; ANI group, n = 8). (B) Effects of protein synthesis inhibition in the ACC (VEH group, n = 7; ANI group, n = 9). Two-way ANOVA followed by a *post hoc *Bonferroni's test revealed a significant effect of time (training vs. test), but not drug (VEH vs. ANI), and no significant time vs. drug interaction when 2 h-STM was assessed (mPFC, time: F_1,34 _= 42.923, P < 0.05; drug: F_1,34 _= 0.888, P > 0.05; time vs. drug: F_1,34 _= 0.907, P > 0.05; ACC, time: F_1,28 _= 45.602, P < 0.05; drug: F_1,28 _= 0.875, P > 0.05; time vs. drug: F_1,28 _= 0.993, P > 0.05). Consistently, the *post hoc *Bonferroni's test revealed that the VEH and ANI groups showed comparable crossover latencies for the 2 h-ST-IA memory (P > 0.05). These results indicate that the inhibition of protein synthesis in the mPFC or ACC did not affect ST-IA memory. Error bars are SEM. Click here for file

Additional file 4 **Effects of inhibiting protein synthesis in the brain regions close to the mPFC or ACC**. (A) Effects of protein synthesis inhibition in the brain regions where cannula tips failed to target on boundaries of the mPFC and ACC (Non-targeted group). This group showed comparable crossover latencies with VEH group (p > 0.05; VEH group, n = 19; Non-targeted group, n = 7). (B) Illustrating cannula tip placements of Non-targeted group. These results indicated that the inhibition of protein synthesis in the brain regions close to the mPFC or ACC did not affect LT-IA memory, suggesting that protein synthesis in the mPFC and ACC is specifically required for the formation of LT-IA memory. Error bars are SEM. Click here for file
