# Supplementary figures and images for: Induction and requirement of gene expression in the anterior cingulate cortex and medial prefrontal cortex for the consolidation of inhibitory avoidance memory
Source: Mol Brain. 2011 Jan 19;4:4. doi: 10.1186/1756-6606-4-4 (PMC3035037; doi:10.1186/1756-6606-4-4)

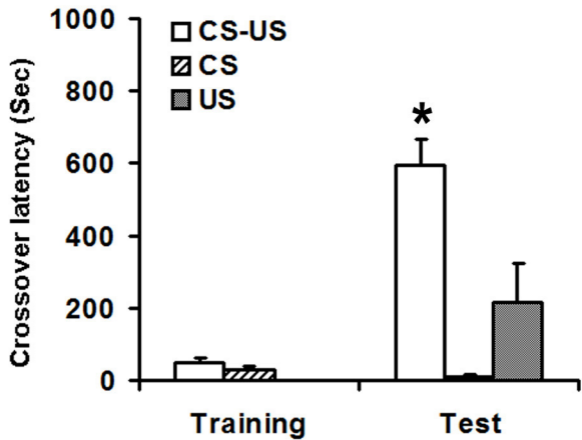

Supplement: Additional file 1 — Crossover latencies of CS-US, CS and US groups. CS and US groups showed significantly shorter crossover latencies than CS-US group during the test at 24 h after training (F3,27 = 22.390, P < 0.05). CS-US, n = 11; CS only, n = 6; US only, n = 6. Error bars are SEM. *P < 0.05 compared with the unconditioned groups. [file 1756-6606-4-4-S1.PDF]

A

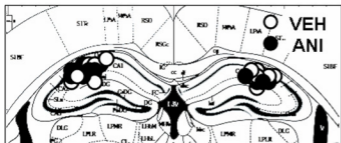

B

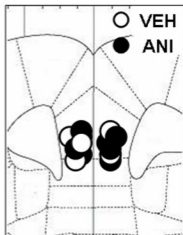

C

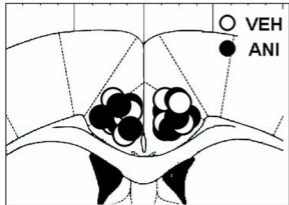

Supplement: Additional file 2 — Illustrating cannula tip placements in the hippocampus, mPFC, and ACC. (A-C) Coronal drawing showing the placement of the cannula tip in the hippocampus (A), mPFC (B) and ACC (C). Only mice with needle tips within the boundaries of the hippocampus, mPFC, or ACC were included in the data analysis. VEH, vehicle; ANI, anisomycin. [file 1756-6606-4-4-S2.PDF]

**A****mPFC**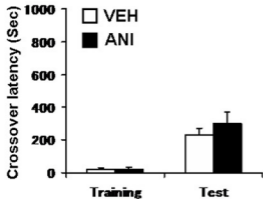**B****ACC**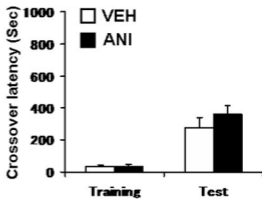

Supplement: Additional file 3 — Effects of inhibiting protein synthesis in the mPFC and ACC on 2 h-STM. (A) Effects of protein synthesis inhibition in the mPFC (VEH group, n = 11; ANI group, n = 8). (B) Effects of protein synthesis inhibition in the ACC (VEH group, n = 7; ANI group, n = 9). Two-way ANOVA followed by a post hoc Bonferroni's test revealed a significant effect of time (training vs. test), but not drug (VEH vs. ANI), and no significant time vs. drug interaction when 2 h-STM was assessed (mPFC, time: F1,34 = 42.923, P < 0.05; drug: F1,34 = 0.888, P > 0.05; time vs. drug: F1,34 = 0.907, P > 0.05; ACC, time: F1,28 = 45.602, P < 0.05; drug: F1,28 = 0.875, P > 0.05; time vs. drug: F1,28 = 0.993, P > 0.05). Consistently, the post hoc Bonferroni's test revealed that the VEH and ANI groups showed comparable crossover latencies for the 2 h-ST-IA memory (P > 0.05). These results indicate that the inhibition of protein synthesis in the mPFC or ACC did not affect ST-IA memory. Error bars are SEM. [file 1756-6606-4-4-S3.PDF]

**A**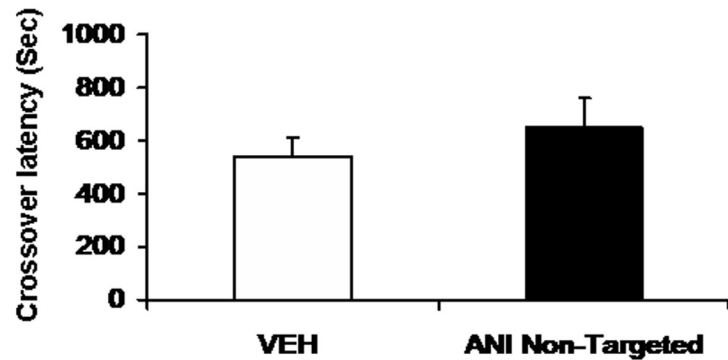**B**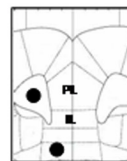

Outside of the  
PL and IL

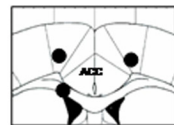

Outside of the  
ACC

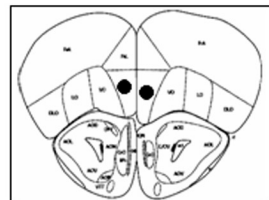

Anterior to the mPFC  
(Bregma +2.58)

Supplement: Additional file 4 — Effects of inhibiting protein synthesis in the brain regions close to the mPFC or ACC. (A) Effects of protein synthesis inhibition in the brain regions where cannula tips failed to target on boundaries of the mPFC and ACC (Non-targeted group). This group showed comparable crossover latencies with VEH group (p > 0.05; VEH group, n = 19; Non-targeted group, n = 7). (B) Illustrating cannula tip placements of Non-targeted group. These results indicated that the inhibition of protein synthesis in the brain regions close to the mPFC or ACC did not affect LT-IA memory, suggesting that protein synthesis in the mPFC and ACC is specifically required for the formation of LT-IA memory. Error bars are SEM. [file 1756-6606-4-4-S4.PDF]
